# The CT and MRI observations of small cell neuroendocrine carcinoma in paranasal sinuses

**DOI:** 10.1186/s12957-015-0475-z

**Published:** 2015-02-15

**Authors:** Qingqiang Zhu, Wenrong Zhu, Jingtao Wu, Hongying Zhang

**Affiliations:** Department of Medical Imaging, Subei People’s Hospital, Medical School of Yangzhou University, No. 98 West Nantong Road, Yangzhou, 225001 China

**Keywords:** Paranasal sinuses, Small cell neuroendocrine carcinoma, X-ray computed tomography, Magnetic resonance imaging

## Abstract

**Background:**

Primary small cell neuroendocrine carcinoma (SNEC) of paranasal sinuses is an extremely rare malignant tumor known for its aggressive clinical behavior.

**Methods:**

Nineteen patients with SNEC in paranasal sinuses by magnetic resonance imaging (MRI) (*n* = 19) and computerized tomography (CT) and MRI (*n* = 18) were retrospectively studied. CT and MRI were undertaken to investigate tumor features.

**Results:**

The lesions were located in the ethmoidal sinus (*n* = 6), maxillary sinus (*n* = 4), and bilateral sphenoid sinus (*n* = 9). All lesions showed a symmetry or ‘pigeon’ pattern in the bilateral sphenoid sinus (*n* = 9). On CT scan, the lesions showed to be isodense (*n* = 3) or mild hyper-dense (*n* = 15). Bone changes included bony absorption or sclerosis (*n* = 3) and moth-eaten bone destruction (*n* = 16). Mild cystic components were visible in five patients with SNEC. There was no evidence of calcification in any of the SNEC tumors. The lesions were isointense on T_1_WI and isointense (*n* = 6) or mild hyper-intense on T_2_WI (*n* = 13). The lesions showed mild or moderate homogeneous enhancement after the administration of a contrast agent. The aggressive nature of the tumors was demonstrated by invasion of adjacent structures, which showed involvement of the nasal cavity (*n* = 17), orbits (*n* = 15), pterygopalatine fossa (*n* = 9), ethmoidal sinus and sphenoid (*n* = 5), clivus ossis occipitalis (*n* = 2), cavernous sinus and internal carotid canal (*n* = 5), optic canal (*n* = 3), jugular fossa (*n* = 2), anterior fossa (*n* = 2), apex partis petrosae ossis temporalis (*n* = 3), meninges (*n* = 2), temporal fossa and infratemporal fossa (*n* = 4), and pharyngonasal cavity and parapharyngeal space (*n* = 3). There was evidence of distant metastasis in five (lung) and one (liver) of the tumors. Fifteen patients (15/19, 78.9%) expired within 5 years of the initial diagnosis, and the other patients are currently still alive.

**Conclusions:**

A tumor exhibiting mild or moderate homogeneous enhancement together with a symmetry or ‘pigeon’ pattern in the bilateral ethmoidal sinus may be considered as specific MRI features.

## Background

Primary small cell neuroendocrine carcinoma (SNEC) of paranasal sinuses is an extremely rare malignant tumor known for its aggressive clinical behavior [1,2]. Moreover, SNECs originating in the paranasal sinuses have been reported to have a poor prognosis [3]. This tumor usually occurs in elderly persons with main complaints being nasal obstruction, epistaxis, loss of visual acuity, exophthalmos, local pain, and rarely tender swelling over the sinuses [4]. Computerized tomography (CT) scan and magnetic resonance imaging (MRI) are essential to assess the size, extent of the tumor, evidence of bone destruction, and infiltration to the orbit or brain [5]. Moreover, CT combined MRI can provide more comprehensive informations in the diagnosis and therapy [6]. Single cases of SNEC were described in the literature, larger groups of patients are scanty [7,8]. The present study deals with 19 cases of SNECs of paranasal sinuses, their clinical presentations, CT and MRI imaging findings, and histopathological diagnosis with review of the literature.

## Methods

### Patient eligibility

A search of pathology records and PACS system identified 19 patients {Chinese, 84% (16/19) of patients were diagnosed by biopsy} with SNEC in paranasal sinuses who were hospitalized at Subei People’s Hospital, Yangzhou, China from January 2006 to September 2014. Eighteen patients underwent CT, and all patients underwent MRI examination. Details of the patients’ age, gender, tumor location, and clinical symptoms are recorded.

### CT imaging technique

All examinations were performed on a multidetector computed tomography (MDCT) scanner (Somatom Definition, Siemens AG, Medical Solutions, Forchheim, Germany). The parameters of the CT scanner are 250 mAs, 120 kVp, a rotation time of 0.75 s, a pitch of 1.204, a 25-cm field of view, a matrix size of 512 × 512, a slice thickness of 1.5 mm, and a detector configuration of 64 × 0.625 mm.

### MR imaging technique

MR examination was performed with a 1.5 T (*n* = 5) or 3.0 T (*n* = 14) MR scanner (Vision or Symphony, Siemens Medical Solutions, Iselin, NJ, USA or Excite Twin Speed, GE Medical Systems, Waukesha, WI, USA) on patient for head imaging. Before contrast injection, standard brain protocol was applied: unenhanced axial T_1_-weighted images, axial, coronal and sagittal T_2_-weighted images, and axial fluid attenuated inversion recovery sequences were obtained. The parameters of the MRI scanner are a 23-cm field of view, a matrix size of 256 × 162, and a slice thickness of 4 mm. T_1_-weighted spin echo (SE) images were obtained in the axial plane (repetition time/echo time (TR/TE), 279/2.3 ms, two excitations). T_2_-weighted fast SE images (TR/TE, 3,118/80 ms, one excitation) and T_2_-weighted short-time inversion recovery (STIR) in the axial and coronal planes were obtained before injecting the contrast material. After the intravenous administration of gadopentetate dimeglumin (Gd-DTPA, Magnevist, 0.1 mmol/kg body weight, injection rate: 1.5 ml/s). Fat-saturated T_1_-weighted SE images were obtained in the axial, coronal, and sagittal planes with the same parameters that were used before Gd-DTPA injection. Eleven cases had time-signal intensity curve (TIC) examination.

### Pathological examination

Pathological specimens were observed by light microscopy and immunohistochemical analysis. All renal tumors were confirmed to be SNEC in paranasal sinuses.

### Imaging analysis and statistics

Two paranasal sinus radiologists analyzed the images together, a process that resulted in a consensus interpretation. The CT and MRI imaging parameters included tumor position and attenuation on unenhanced CT scan, MRI signal, invasion of adjacent structures, the degree of enhancement on MRI scan, and so on. The enhancement pattern of the tumor was classified as homogeneous or heterogeneous.

## Results

The study included 19 patients (15 females and 4 males) with SNEC in paranasal sinuses. The mean age at diagnosis was (46.7 ± 7.6) years (range from 26 to 63 years). Headache, vision loss, hyposmia, yellow nasal discharge, and exophthalmos were found in 17, 12, 11, 11, and 7 out of 19 patients, respectively.

**Figure 1:**
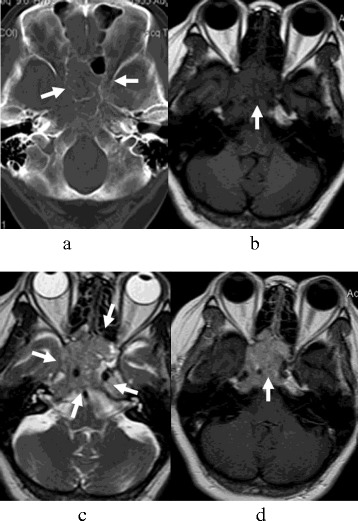
**SNEC of paranasal sinuses in a 41-year-old man (a-d).** The lesion was symmetrical, and the size was about 5.8 cm × 5.7 cm × 4.3 cm. **(a)** CT image showed worm-eaten bone destruction in sphenoid sinus, anterior cranial fossa, and orbital apex; however, bone contours still could be seen. **(b)** T_1_-weighted MR image demonstrated isointensity. **(c)** T_2_-weighted MR image demonstrated isointense together with a ‘pigeon’ pattern. **(d)** Contrast-enhanced T_1_-weighted MR image demonstrated a moderate heterogeneous enhancement mass, which showed involvement of the pharyngonasal cavity, orbital apex, pterygopalatine fossa, sella, cavernous sinus, internal carotid canal, and jugular foramen.

**Figure 2:**
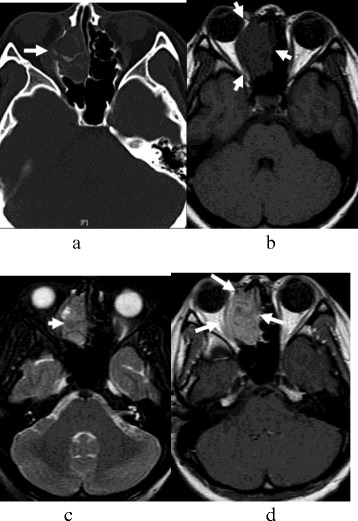
**SNEC of paranasal sinuses in a 53-year-old man (a-d).** The tumor size was about 4.3 cm × 4.1 cm × 3.1 cm. **(a)** CT image showed worm-eaten bone destruction in the right ethmoidal sinus and fossa orbitalis; however, bone contours still could be seen. **(b)** T_1_-weighted MR image demonstrated isointensity. **(c)** T_2_-weighted MR image demonstrated isointense mixture. **(d)** Contrast-enhanced T_1_-weighted MR image demonstrated a mild heterogeneous enhancement mass, which showed involvement of the pharyngonasal cavity and fossa orbitalis.

The lesions were located in the bilateral sphenoid sinus (*n* = 9, Figure 1), ethmoidal sinus (*n* = 6, Figure 2), and maxillary sinus (*n* = 4). All lesions showed a symmetry or ‘pigeon’ pattern in the bilateral sphenoid sinus (Figure 1).

On CT scan, the lesions showed to be isodense (*n* = 3) or mild hyper-dense (*n* = 15). Bone changes included bony absorption or sclerosis (*n* = 3) and moth-eaten bone destruction (*n* = 16). Mild cystic components were visible in five patients with SNEC. There was no evidence of calcification in any of the SNEC tumors.

The lesions were isointense on T_1_WI and isointense (*n* = 6) or mild hyper-intense on T_2_WI (*n* = 13). The lesions showed mild or moderate homogeneous enhancement after the administration of a contrast agent. TIC showed plateau type in 11 cases. The aggressive nature of the tumors was demonstrated by invasion of adjacent structures, which showed involvement of the nasal cavity (*n* = 17), orbits (*n* = 15), pterygopalatine fossa (*n* = 9), ethmoidal sinus and sphenoid (*n* = 5), clivus ossis occipitalis (*n* = 2), cavernous sinus and internal carotid canal (*n* = 5), optic canal (*n* = 3), jugular fossa (*n* = 2), anterior fossa (*n* = 2), apex partis petrosae ossis temporalis (*n* = 3), meninges (*n* = 2), temporal fossa and infratemporal fossa (*n* = 4), and pharyngonasal cavity and parapharyngeal space (*n* = 3). There was evidence of distant metastasis in five (lung) and one (liver) of the tumors.

Macroscopic examination reveals sheets, cords, or ribbons of small cells with little cytoplasm. Scattered areas of necrosis may be observed, and the typical crush artefact of neoplastic cells is often visible. Hemorrhage (hemosiderin deposition) was visible only in two patients with SNEC. Immunochemistry staining demonstrated that Syn, CK, CgA100, and NSE were positive in 18, 17, 16, and 16 cases, respectively.

In this study, all of the 19 patients with SNECs underwent surgery, radiotherapy, or chemotherapy, of which all patients were able to be followed up from 13 to 167 months. Fifteen patients (78.9%) expired within 5 years of the initial diagnosis, and the other patients are currently still alive.

## Discussion

SNEC occurs mainly in the lungs and accounts for approximately 20% of primary lung carcinomas [9]. Less than 270 cases of head and neck SNEC have been published until 2006 March including 69 cases of SNEC in nasal and paranasal sinuses in the world literature [10,11]. Similar to SNEC of the lung, SNEC of the nasal and paranasal cavities has demonstrated aggressive clinical behavior and a poor prognosis, with fast tumor expansion, early local recurrence, and widespread dissemination [12].

SNEC in paranasal sinuses has been described to affect primarily adults without gender differences [13]. The average age of incidence is about 41.5 to 55.0 years old (10 to 79 years), and the common sites of SNEC are ethmoid sinus and maxillary sinus [14]. The age of our group is about 26 to 63 years old, which is similar to prior reports [15].

No particular risk factor for the tumor occurring in elderly patients has been identified [15]. Clinical presentations include nasal obstruction, epistaxis, facial mass, or facial pain [16]. The most frequent sites for distant metastases are the lungs, liver, and bone [17]. Our results showed distant metastases in the lung (*n* = 5) and liver (*n* = 1), which is coincidence with the prior reports.

Surgery, radiotherapy, or chemotherapy therapeutic methods either alone or in combinations are used by various head and neck oncologists [18]. A group of oncologists favored surgery followed by radiotherapy [19]. However, more recent studies have claimed that neoadjuvant chemotherapy with cisplatin and etoposide and high-dose proton-photon radiotherapy is a successful treatment approach for SNEC patients in paranasal sinuses [20]. In this study, all of the 19 patients with SNECs underwent surgery, radiotherapy, or chemotherapy, of which all patients were able to be followed up from 13 to 167 months. Fifteen patients expired within 5 years of the initial diagnosis, and the other patients are currently still alive, which is coincidence with the prior reports.

Calcification (0/19) and hemorrhaging (2/19) are rare in SNEC, and accordingly, these features were not present radiologically or pathologically in the current study. In addition, bony destruction has been a common imaging feature of SNEC in previous studies. In the current study, bony erosion was frequently observed in paranasal sinuses and sclerosis was rare [21]. Radiological examination is important to determine the extent of local tumor invasion and distant metastasis [22]. CT or MRI imaging of the paranasal sinuses are more useful than conventional radiography when assessing the extent of local invasion of the tumor and are better for the planning of further treatment.

SNEC in paranasal sinuses shows rare necrosis or hemorrhage on pathology, which is coincidence with the homogeneous signal on T_2_WI or enhanced T_1_WI [23]. Primary sphenoid sinus tumors account for 2% of all paranasal sinus tumors. In addition, the signals of these tumors were homogeneous isodense or mild hyper-dense on CT, which could not be interpreted as hemorrhaging [24]. This indicated that the tumor contained neuroendocrine grana and that the neuroendocrine cells may have grown along the ethmoidal cells, which may have resulted in the existence of this mucus in the SNEC.

In our study, nine cases distributed a symmetry or ‘pigeon’ pattern in the bilateral ethmoidal sinus. The characteristics were only found in the SNEC and may be considered as specific MRI features. A tumor exhibits features, for example, the lesions were located in the sphenoid sinus and distributed a symmetry or ‘pigeon’ pattern. MRI showed that the tumor grows invasively, with mild or moderate homogeneous enhancement, except for the typical imaging findings of squamous cell carcinoma, lymphoma, and adenoid cystic carcinoma, so we considered the possibility of SNEC [25].

The differential diagnosis of SNEC in paranasal sinuses includes inverted papilloma (IP), squamous cell carcinoma (SCC), adenocarcinoma, adenoid cystic carcinoma (ACC), lymphoma, and olfactory neuroblastoma (ONB). Those tumors have similar radiologic findings such as soft tissue mass, bony destruction, and different patterns of enhancement. IP has a typically ‘lobulated’ or ‘cerebriform’ configuration on CT and MRI and may contain hyper-dense foci [26]. SCC of the sinonasal cavities commonly occurs in men, with a peak incidence in the sixth and seventh decades. Bony erosion on CT and intermediate signal intensity on T_2_WI are the hallmarks. ACC affects a broad age range, but it is uncommon before the age of 20 years and the peak incidence is in the fifth decade. The imaging features of sinonasal ACC may be observed with a combination of bony erosion and sclerosis on CT and intermediate or high signal intensity on T_2_WI. Lymphoma usually demonstrates bony erosion or infiltration of the sinonasal cavities and orbits without calcification [27]. It exhibits intermediate signal intensity on T_1_WI and T_2_WI and homogeneously contrast enhancement on postcontrast CT or MRI. ONB is an aggressive neuroendocrine tumor located highly in the nasal cavity. It affects a wide range with peaks in the second and fifth or sixth decade. Peripheral areas of cystic degeneration and calcific foci are radiologic features associated with ONB.

The present study had certain limitations. Firstly, a small number of patients were included owing to the rarity of SNEC, and secondly, 84% (16/19) of patients were diagnosed by biopsy. Therefore, further multicenter cooperation on the radiological diagnosis of SNEC is required.

## Conclusions

In conclusion, SNEC is a rare and aggressive malignancy. CT or MRI accurately demonstrates the location and extent range of SNEC; however, SNEC may exhibit certain imaging features that are similar to other tumors in the head and neck regions. The present study indicated that a tumor exhibiting mild to moderate homogeneous enhancement together with a symmetry or ‘pigeon’ pattern in the bilateral ethmoidal sinus may be considered as specific MRI features.
